# Could secondary flows have made possible the cross-strait transport and explosive invasion of *Rugulopteryx okamurae* algae in the Strait of Gibraltar?

**DOI:** 10.1371/journal.pone.0285470

**Published:** 2023-05-23

**Authors:** Jesús García-Lafuente, Irene Nadal, Simone Sammartino, Nathalie Korbee, Félix L. Figueroa

**Affiliations:** 1 Physical Oceanography Group, Instituto de Biotecnología y Desarrollo Azul (IBYDA), Universidad de Málaga, Málaga, Spain; 2 Physical Oceanography Group, Instituto de Ingeniería Oceánica (IIO), Universidad de Málaga, Málaga, Spain; 3 Department of Ecology and Geology, Instituto de Biotecnología y Desarrollo Azul (IBYDA), Universidad de Málaga, Málaga, Spain; University of Guam, GUAM

## Abstract

Presently, the Strait of Gibraltar is undergoing an unprecedented invasion of the alien alga *Rugulopteryx okamurae* of North Pacific origin. According to the scarce literature, the algae first settled in the south shore, probably following commercial exchanges with French ports where it was accidentally introduced together with Japanese oysters imported for mariculture. There is no certainty, however, that the algae first colonized the south shore of the Strait and, from there, spread to the north. It could well have been the opposite. Whatever the case, it spread all over the Strait and surrounding areas with amazing rapidity. Human-mediated vectors (algae attached to ship hulls or fishing nets, for example) can be behind the spread from the shore initially settled to the algae-free shore on the opposite side. But it could also have happened by means of hydrodynamic processes without direct human intervention. This possibility is assessed in this paper by revisiting historical current meter profiles collected in the Strait of Gibraltar searching for secondary cross-strait flows. All the stations present an intermediate layer of northward cross-strait velocity near the interface of the mean baroclinic exchange along with a surface layer above of southward velocity, whose lower part also overlaps the interface zone. The first one would back the south-to-north transport of algal fragments, the second one, the north-to south. In both cases, algae must reach the depth of the interface. The vertical velocity field in the area, which far exceeds the small sedimentation velocity of the algae, allows their vertical displacements throughout the water column. Its endurance to survive under the weak or no light conditions that will prevail during the cross-strait transport and its capability of reactivating the metabolism after this unfavorable period, offers chances for colonizing the opposite shore. Therefore, the propagation of the algae by hydrodynamic processes, without human intervention, cannot be ruled out.

## Introduction

**Fig 1:**
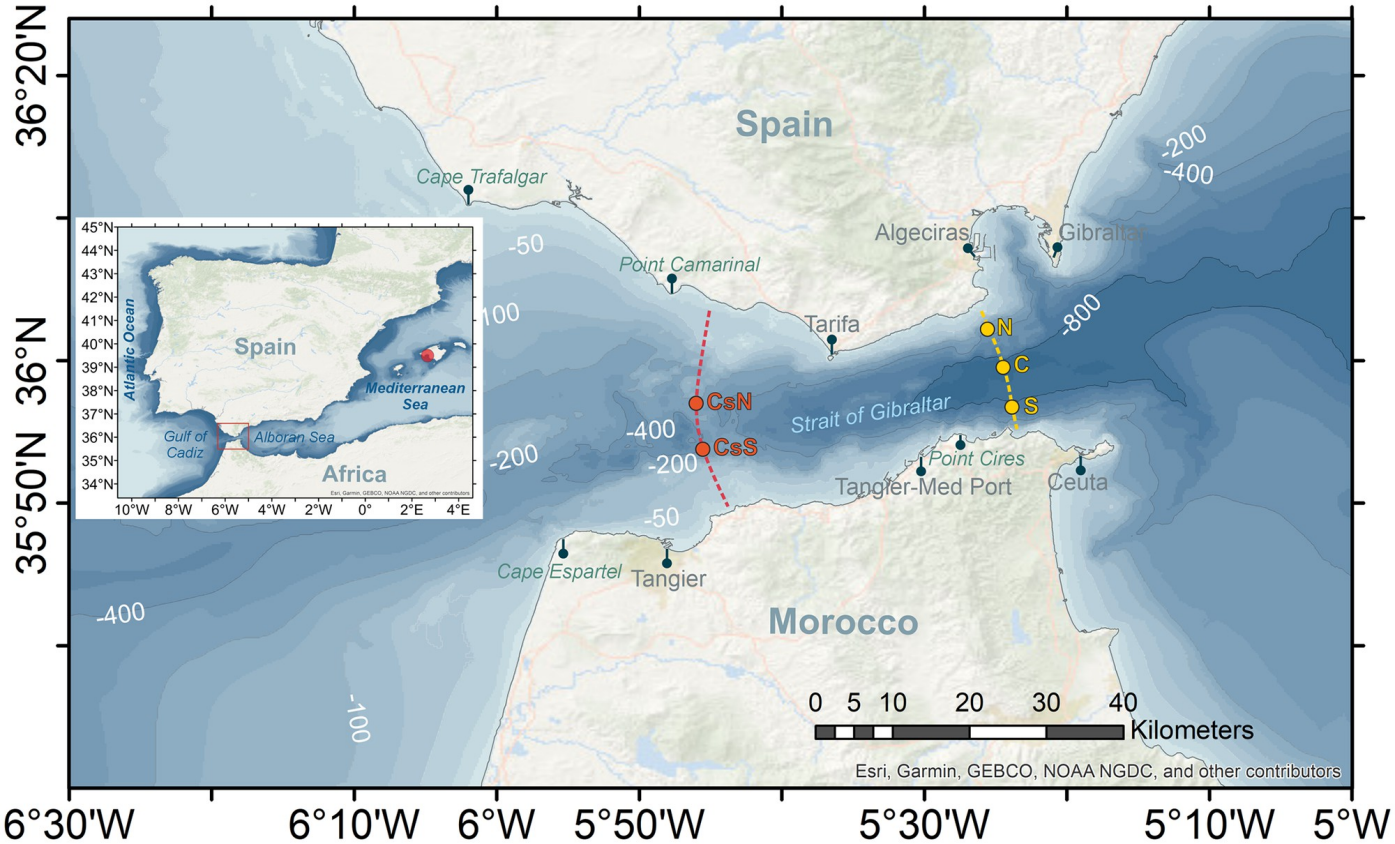
Map of the Strait of Gibraltar showing bathymetric features and locations and geographic sites mentioned in the text. Dots labelled N, C, and S in the eastern part and CsN and CsS in Camarinal section are the stations where current meter data used in this study come from. Red dot in the inset indicates Palma de Mallorca in the Balearic Islands. Sources: Esri, GEBCO, NOAA, National Geographic, DeLorme, HERE, Geonames.org, and other contributors. Maps were created using ArcGIS^®^ software by Esri. ArcGIS^®^ and ArcMap^™^ are the intellectual property of Esri and are used herein under license. Copyright Esri. All rights reserved. For more information about Esri^®^ software, please visit www.esri.com.

**Fig 2:**
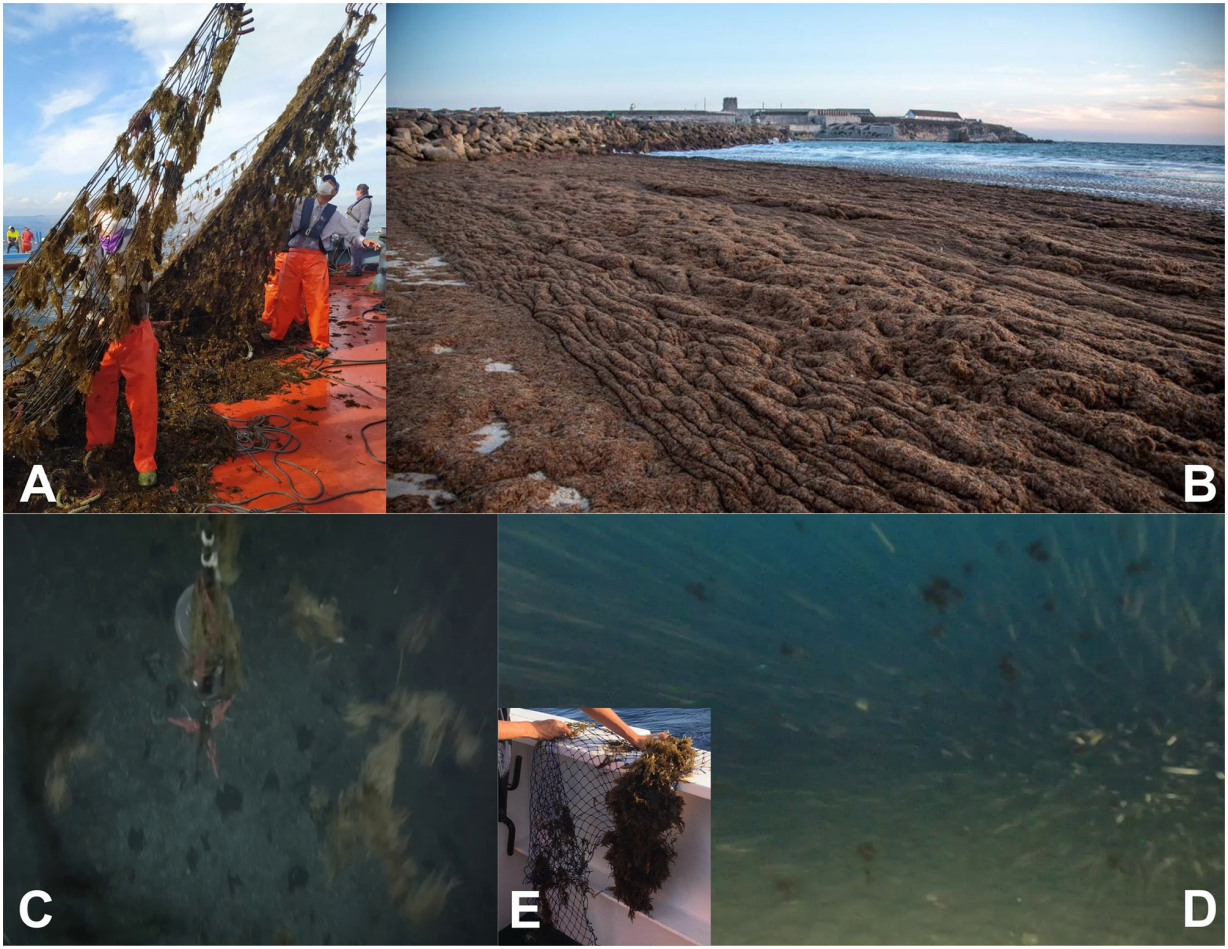
A) Tuna fishing net (Almadraba) pulled from the sea in Tarifa nearly covered with *Rugulopteryx okamurae*. B) Massive *Rugulopteryx okamurae* beaching nearby Tarifa. C) Frame taken from a video recorded by a Remotely Operated Vehicle during a rescue operation of a mooring line off Cape Espartel (see Fig 1) showing thalli of *Rugulopteryx okamurae* drifted by the Mediterranean outflow. Sea floor (identified by the grey spots in the background) is at 360m depth. D) Frame taken from a video recorded by a camera attached to a trawling net showing several fragments of *Rugulopteryx okamurae*. The fishing vessel was working to the northeast, but near of, the eastern limit of the SoG and the videocamera was at 120m depth. E) Fragments of *Rugulopteryx okamurae* attached to scientific equipment deployed at 350m depth off Espartel which were collected when the instruments were brought to the surface for maintenance.

Since the mid-2010s, the coasts around the Strait of Gibraltar (SoG, hereinafter) and neighboring basins (Fig 1) are suffering from a drastic invasion of the algae *Rugulopteryx okamurae* [1], a brown macroalga belonging to the Dictyotaceae family (Dictyotales order), original from the northwestern Pacific Ocean [2]. The alga was detected in the small Mediterranean lagoon of Thau, southwest of France, in year 2002 for the first time in Europe [3] where, according to these authors, it was probably introduced along with Japanese oysters imported for mariculture purposes. In year 2015 the alga was found off Ceuta in the south coasts of the SoG [4–7] where it could have arrived following commercial exchanges with Tangier-Med port (Fig 1). Leisure boats and their marinas have been also shown to be important components of the hub-and-spoke model of invasion in coastal systems [8]. On the other hand, algae could have already been present on the north coast at that time and not have been identified due to its resemblance to other algae already established in the area, although a benthic community monitoring program at some fixed sentinel stations established on the north coast did not detect algae up until 2016 [1]. In spite of this fact, which gives weak support to the southern pathway, the precise route of entry still remains uncertain. Regardless this uncertainty, the point is that, in few years, the alga has occupied the illuminated rocky seafloor of large extended coastal areas in both shores of the SoG and adjacent basins [1], featuring an aggressive invasion with no precedents in European coastal waters (Fig 2A and 2B). Northern Africa and the southern Iberian Peninsula are the most intensely affected area, but it continues expanding towards the west and east with the threat of monopolizing the sea rocky bottom to the detriment of photophilous resident biota [1, 9]. Currently, the exotic alga has colonized the Marsella coast [10] and even the Azores islands [11]. More worrisome is the risk pointed out by [12] that it could cover the whole Mediterranean coasts.

The explosive spreading would be linked to favorable environmental conditions, since the photic zone in the area gathers suitable year-round temperature for growth and reproduction of this subtropical species [1, 13], but also to the fact that broken thalli serve as seeding population, since their specimens present vegetative propagules [4, 14]. A remarkable fact to this regard is that thalli and propagules are actually found drifting not only in the illuminated zone where they are also fixed to the bottom but also at practically any depth of the SoG (Fig 2C–2E).

The plausible hypothesis that algae first settled in a shore of the SoG, likely the south one according to the scarce available literature, poses the question of how did it spread to the other shore. Different mechanisms could be behind the spreading. The first and more plausible one is linked to the heavy ship traffic in the area, which includes continuous north-to-south ferry and other commercial ships crossing the Strait. The alga shows huge capability to colonize any type of hard substrates, not only rocky seafloor but glass, ceramic, iron, tires, etc. [15], so the eventual crossing attached to ship hulls, fishing nets, including scientific sampling nets [16], or other human-mediated vectors as the mentioned recreational boats [8] cannot be ruled out. Neither ballast waters from remote shipments can be discarded [17, 18]. Another possibility, however, is that the algae managed to cross the SoG taking advantage of hydrodynamic processes without human intervention. Such possibility is revised in this paper. Interestingly, it would be also applicable to pelagic larvae and propagule of other marine species that last for weeks in the pelagic realm. This opens new hypothesis about transport mechanisms between Lusitanian and Mauritanian biogeographical regions, which could eventually be testable by applying molecular techniques among populations.

## Possible mechanisms for cross-strait connection

The SoG holds a well-known two-way (baroclinic) exchange necessary to compensate for the freshwater and buoyancy losses in the Mediterranean basin. It results in intense, zonal-oriented currents: an eastward Atlantic inflow into the Mediterranean Sea at the surface, and a subsurface Mediterranean outflow towards the Atlantic Ocean beneath (see [19] or [20], for instance). Under such a system of currents, the spreading of alga and further substrate colonization in west-east direction would be achievable relatively easily, but it will not be that easy in the cross-strait direction. The intense along-strait currents behave as hydrodynamic barriers for cross-strait transport [21] that, moreover, would carry propagules out of the dimensions of the SoG well before they have chances to get to the opposite shore.

**Fig 3:**
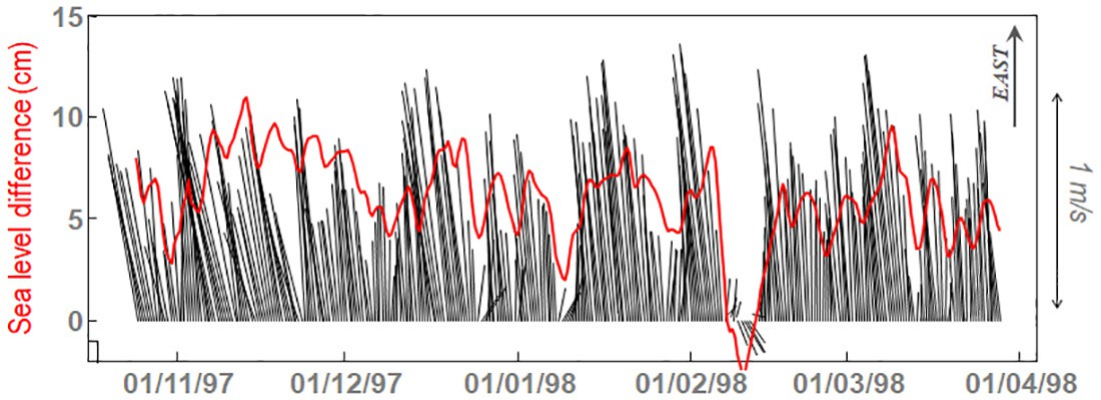
Black lines: Stick diagram of the velocity (scale on the right) recorded at 50m depth in site “C”, station C2 (Fig 1, Table A in S1 File). Vectors are oriented with reference to the East, indicated in the upper-right corner. Red line is the sea level difference between south (Ceuta, see Fig 1) and north (Algeciras) shores. Positive values indicate northward sea surface downslope (higher sea level in the south) as predicted by the geostrophic adjustment of an eastward surface current. (Adapted from [34]).

There is, however, a loophole for this dynamic constriction, which is the necessary vanishing of the along-strait current at the depth of the interface between the Atlantic and Mediterranean layers. Should exist a cross-strait secondary flow at this depth, even if weak, the chances to successfully cross the Strait would be no null. Secondary flows are linked to rotation and friction with the solid boundaries, which are the ingredients of Ekman boundary layers [22, 23]. The along-strait inflow and outflow are density driven flows, as there is no geostrophic balance (pressure gradients balanced by Coriolis force) in the along-strait equation. However, the earth rotation acting upon them, forces a pressure gradient that leads to geostrophic cross-strait balance [19, 20, 24], a pattern known as semi-geostrophy or semi-geostrophic flows. Fig 3 shows the good agreement between that pressure gradient, as given by the sea level difference between south and north shores, and the along-strait velocity in the Atlantic layer at the eastern SoG.

**Fig 4:**
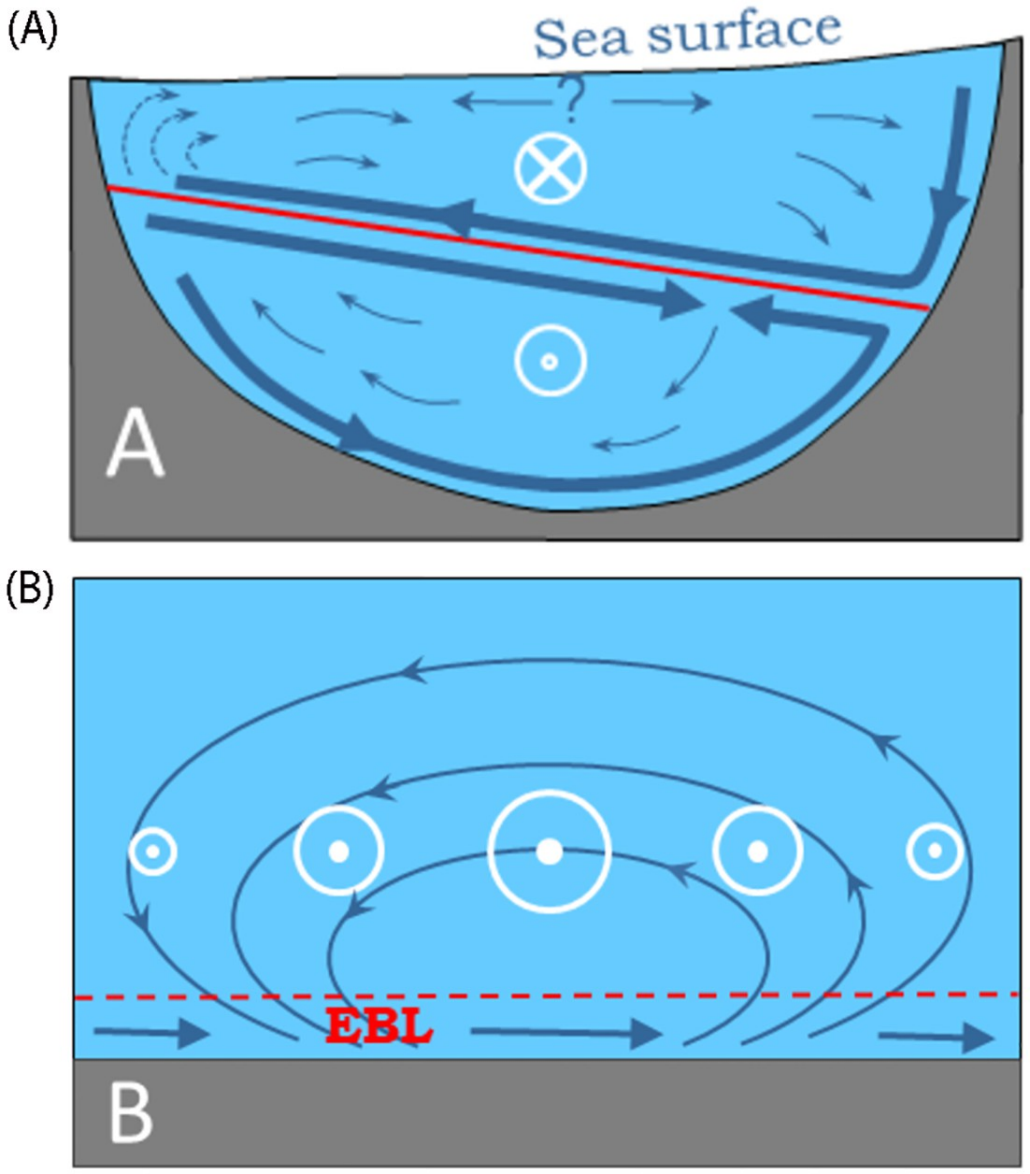
A) Schematic of a two-layer exchange through a channel of half-circular geometry, adapted from Johnson and Ohlsen (1994). Encircled white cross (dot) indicates flow into (out of) the page, thus resembling the exchange through the SoG as observed from the Atlantic looking to the Mediterranean, with the north (south) shore in the left (right). Northward downslope of the free surface and southward downslope of the interface (solid red line) are disclosed. Thick arrows indicate the solid boundary and interfacial boundary Ekman layers, whereas thin arrows illustrate the return flow in the interior (secondary circulation). B) Sketch of the interior ageostrophic circulation (thin arrows) driven by the convergence and divergence of the Ekman transport (horizontal thicker arrows) in the Ekman boundary layer (EBL, dashed red line) beneath a spatially-variable jet, represented by the (size) of the white encircled dots. Since outside of the EBL the flow is geostrophic (water moves along isobars) the secondary circulation represented by the thin arrows crosses the isobars and is therefore ageostrophic or out of geostrophic balance.

The semi-geostrophic balance tends to accumulate water of the surface layer in the south coast of the SoG to build up the pressure gradient, a process that favors the north-to-south transport within this layer. Near the lateral boundary the geostrophically-balanced current is reduced by friction, so that the pressure gradient is able to drive cross-isobar ageostrophic flows (see Fig 4 and caption there) and generate a secondary cross-strait circulation [25, 26]. Laboratory experiments of two-layer exchange through channels of half-circular cross section [27] suggest that this flow is partially driven by the dynamics of Ekman boundary layers, where the net Ekman transport is to the left of the jet that induces the layer, looking downstream (Fig 4A). These authors suggest the existence of robust interfacial Ekman layers contributing to the secondary flow, although the behavior of these layers in the laboratory experiments was more complicated than expected as they did not extend all the way to the lateral boundaries, but apparently converged somewhere by the central part.

On the other hand, a spatially-variable jet flowing over the seafloor causes convergence and/or divergence in the bottom Ekman boundary layer (Fig 4B), in the same manner as a spatially-changing wind stress over the sea surface drives downwelling/upwelling in the open ocean. The jet forces an interior across-jet flow, sketched by the thin blue lines in Fig 4B that, in addition to slightly diminishing the jet strength via the Coriolis force (spin-down process; [23], it facilitates across-jet transport of particles. Near-bottom Mediterranean outflow in the SoG shares spatial characteristics with this idealized jet [28] for which it would provide chances for south-to-north connections in the present case.

The previous discussion has assumed a steady state exchange, which is far from being the case in the SoG. Tidal currents are strong enough to reverse the mean currents at semidiurnal time-scales [19, 28, 29] and, therefore, to cancel the intense flows during short time intervals. A first question is whether tidal dynamics could offer opportunity windows of diminished east-west flows for achieving north-south transport in a complete way. Tidal ellipses (over a tidal cycle, the tips of the tidal velocity vector trace out an ellipse called tidal ellipse) tend to have its major axis aligned with the shoreline and, therefore, its minor axis characterizes the cross-strait tidal velocity. Ellipses are highly polarized [30] with minor axis less than 10 cm·s^-1^ in all cases. A periodic velocity of this size causes periodic displacements of 1.4 km at semidiurnal frequencies, twice this value at diurnal ones, which are clearly insufficient to achieve a successful crossing. However, the shoreline orientation of the SoG changes noticeably from place to place. In the north, the shore veers almost 90° around Tarifa, changing orientation from NW-SE in the west to SW-NE in the east (Fig 1). Something alike happens in the south around Punta Cires (Fig 1). These important changes of orientation cause local misalignments of the ellipses that leave them partially oriented in the cross-strait direction [31]. Tidal currents around such singular sites would then contribute to eventual cross-strait transport more efficiently.

Another potential mechanism is the interaction of the total current with the lateral boundaries. It mainly entails the surface layer, which experiences the effect of abrupt changes of the shoreline orientation more than the Mediterranean layer, where bathymetry hardly changes orientation (Fig 1). [31] or [32], using Lagrangian trajectories of passive tracers computed from the output of a numerical model, show cross-strait intrusions of the tracer in the neighborhood of these particular sites, opening new chances for a successful north-south connection. The mechanism is similar to the previous one, except for the fact that it deals with total current, not only tidal ones.

A detailed investigation of whether these processes can eventually lead to a successful hydrodynamic connection between the opposite shores of the SoG should be carried out by means of high spatial resolution advection schemes coupled to fully 3D, non-hydrostatic numerical models. Previously to such numerical effort, however, searching for observational evidences of cross-strait flows in existing records is an interesting exercise that can assist in future research. This is the objective of this work, which analyses different current meter data collected in the SoG to assess the potential of the recorded currents as vectors for the cross-strait spreading of the *Rugulopteryx okamurae* algae in the area.

## Summary of current meter observations and other data sets

Velocity observations come from three different sources: i) point-wise current meter data collected at points N, C, and S in the eastern SoG (see Fig 1) in the second half of 1990’s decade within the frame of CANIGO project, which have been already employed in several publications (e.g. [20, 33–35]), ii) Acoustic Doppler Current Profiles (ADCP) collected in year 2013 in the main sill of Camarinal (points CsS and CsN in Fig 1) used to investigate high frequency phenomena caused by flow-topography interaction [36], and iii) recent ADCP collected at point CsS (Fig 1) in years 2020–2021, which have not been used yet. Annex A provides more information about locations, time coverage and depths of all these observations.

Atmospheric pressure is provided by Puertos del Estado, Spain, (https://portus.puertos.es) for the period August 2020 –July 2021 at a 1-minute sampling rate in the buoy of Palma de Mallorca (see Fig 1), taken as a representative of the Western Mediterranean basin. Wind data in the sub-region of the SoG during the same time period have been retrieved from the ERA5 reanalysis model [37], which is provided by the Copernicus Climate Change Service (C3S) (https://climate.copernicus.eu) with 1-hour temporal and ∼25 km spatial resolutions.

**Fig 5:**
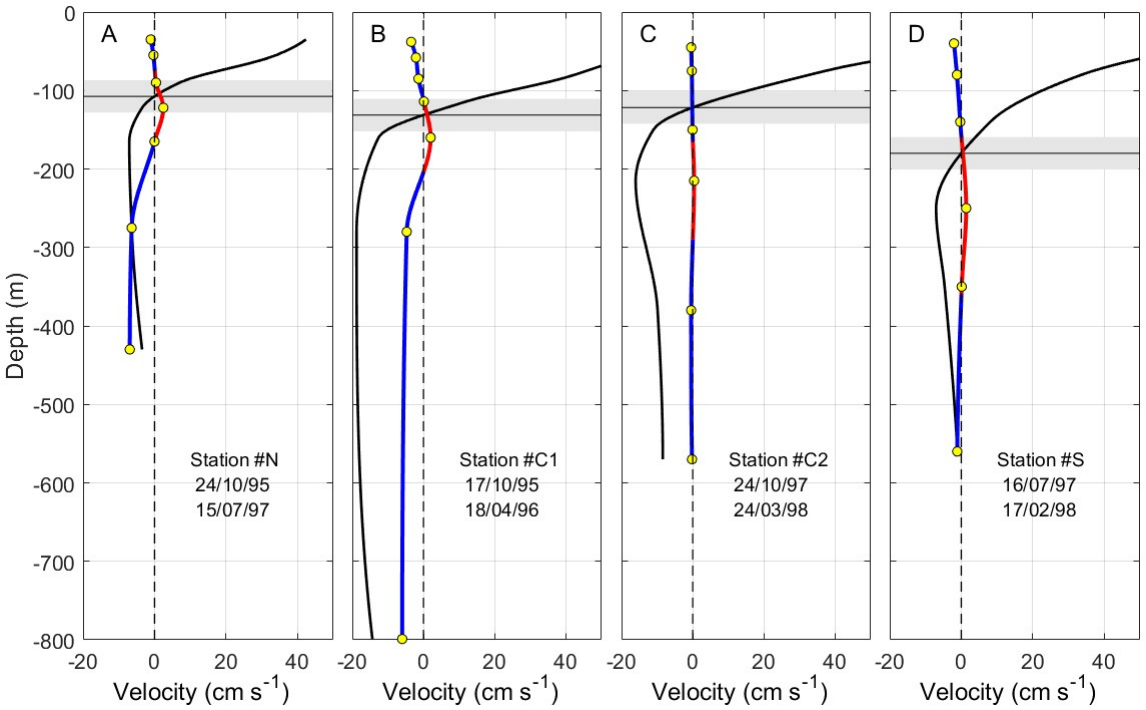
Time-average cross-strait (blue and red line) and along-strait (black line) velocity as a function of depth in the stations of the eastern section. Panel A corresponds to site N, panels B and C are for site C in two different periods, and panel D is for site S (see Fig 1 and Annex A for details). Horizontal black line is the depth of null time-average along-strait velocity, that is, the interface that separates inflow and outflow (notice the greater depth towards the south, in agreement with the sketch of Fig 4A). Shaded rectangle indicates ±std (standard deviation) of the subinertial fluctuations of this interface estimated in station CsS2 (see text for details). In all panels, solid lines are interpolated profiles from observations, whose depths are indicated by yellow circles. The depth range of positive (northwards) cross-strait velocity is marked in red. The ±1 std interval of this velocity is indicated by light-blue thin lines.

**Fig 6:**
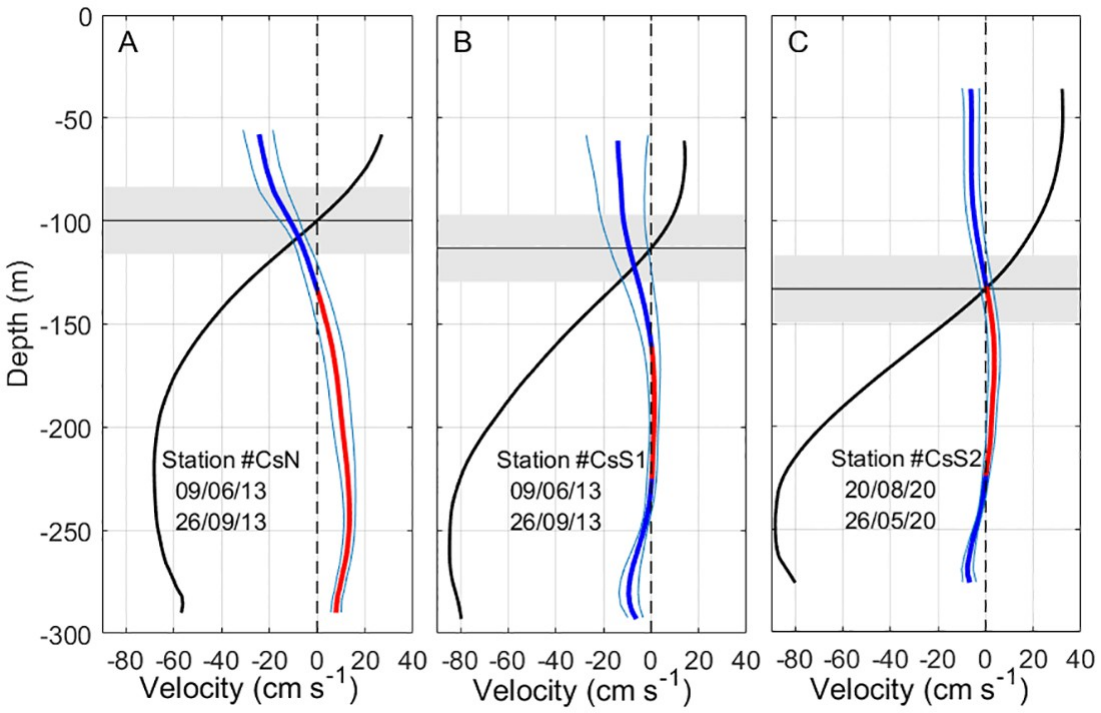
Same as Fig 5 for Camarinal sill section. Panel A is for site CsN and panels B and C are for site CsS in two different periods (see Fig 1 for locations). The profiles are directly computed from ADCP observations (no interpolation required) and circles indicating instrument depths do not apply.

The available current meter time-series have been projected into a rotated Cartesian system aligned with the axis of the SoG (rotated 20° anticlockwise respect the east-north reference system). The *y*-component of the velocity in the new system is the cross-strait velocity, positive northwards, and the *x*-component is the along-strait velocity, positive to the east. The time-average of both velocity components as a function of depth is plotted in Fig 5 for the velocity series collected at the eastern section and in Fig 6 for the series collected at Camarinal sill.

**Fig 7:**
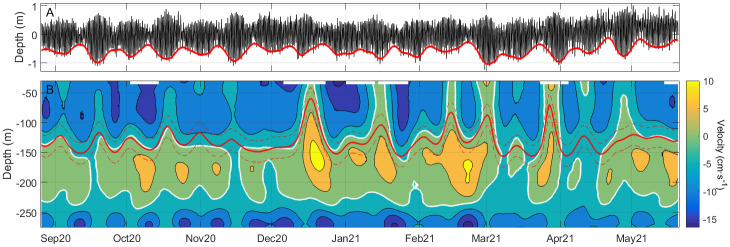
A) Pressure oscillations (converted to meters of water column) recorded by the pressure sensor of the ADCP after removing the mean pressure. The spring-neap tidal cycle is easily recognizable and has been made clearer by the smoothed low-water envelope represented by the thick red line. B) Contours (every 5 cm·s^-1^) of the subinertial cross-strait velocity. Reddish colors indicate positive (northwards) velocity whereas bluish tones are for negative, the white line being the contour of cross-strait null velocity. Solid red line indicates the depth of along-strait null velocity (interface) flanked by two dashed red lines at ±1 std of the mean interface depth. All time series correspond to station CsS2 in Camarinal sill.

The surface of instantaneous null velocity, which is the intuitive interface separating inflow and outflow, exhibits large tidally-driven oscillations. Even more, it often disappears because the entire water column moves in one direction during parts of the tidal cycle [19, 20]. Only after removing tidal motions (by a low-pass filtering), this surface can be tracked, although it undergoes noticeable oscillations (Fig 7B) of meteorological origin [24, 33] that can exceptionally lead to its collapse during short intervals [34].

A mean local interface has been defined as the time-averaged depth of the surface of null velocity obtained from the vertical profiles of velocity at each station. The horizontal black lines in Figs 4 and 5 show the interface depth along with a confidence interval, which shows up as a shaded rectangle. This interval has been taken as the standard deviation of the subinertial fluctuations of the interface observed at station CsS2 (cf. Fig 7). The reason for choosing station CsS2 is the high spatial resolution provided by ADCP observations, necessary for the accurate determination of the null-velocity depth, and the length of the series, the longest of the ADCP stations (see Table B in S1 File).

## Assessment of the possible cross-strait transport

### Observation-based cross-strait mean secondary circulation

The successful algae spreading between both shores depends on the cross-strait current, as the floating spores or seaweed fragments eventually torn from the substrate behave as passive nearly neutrally-buoyant particles (see next Section). However, the success is mediated by the along-strait current, which can displace those particles beyond the open boundaries of the SoG before they are transported from one shore to the other. A favorable coupling between both velocity components is thus necessary.

All the profiles in Figs 4 and 5 show a mid-depth layer of positive cross-strait velocity flanked by negative layers on the surface and at depth, except for CsN site (Fig 6A), where the deeper part is lacking. Accordingly, south-to-north connection would be feasible within the intermediate layer (red line in profiles), whereas north-to-south transport could take place within either the surface or the deep layer (in the eastern SoG, in this case). Figs 4 and 5 show values of cross-strait velocity of around 5 cm·s^-1^ (4 km·day^-1^) or less in the intermediate and deep layers, thus requiring about 4 days at least to cross the SoG at its narrowest section (∼14 km) and longer through other sections. Along-strait mean velocity in the Mediterranean layer changes with depth and geographical location. In its core, the velocity increases from few tens of cm·s^-1^ in the eastern part to nearly 1 m·s^-1^ in the west (Figs 4 and 5). Even assuming a moderate spatial mean value of 40 cm·s^-1^, the along-strait displacement undertaken in 4 days is close to 150 km, well above the dimensions of the SoG. The same or even more unfavorable considerations apply to the surface layer. Therefore, only by avoiding the core of greater velocities and remaining close to the interface could the algae have chances to cross the SoG.

According to Figs 4 and 5, the last condition discards connectivity within the deeper layer and reduces the chances to the upper and intermediate layers. South-to-north (north-to-south) transport could be achieved in the depth range where the red (blue) profile in Figs 4 and 5 overlaps the shaded grey rectangle. The depth range of positive cross-strait velocity overlaps this rectangle more clearly in the eastern part (red lines in Fig 5), suggesting that this section of the SoG gathers better conditions for successful south-to-north connection within the intermediate layer. The contrary would happen in Camarinal sill (Fig 6) where north-to-south transport in the upper layer is favored.

### Time variability of the cross-strait secondary circulation

The previous description based on time-average values of the velocity must be revised when subinertial variability (from days to few tens of days) is considered. Fig 7B shows contours of the cross-strait velocity after filtering the series with a low-pass filter of 3-day cutoff period that removes tides. The depth of the interface of null along-strait velocity together with the ±1 std interval (red lines) have been superposed to the contours. Fig 7A has been included to disclose the fortnightly spring-neap tidal cycle that modulates the semidiurnal tides.

The intermediate layer of positive cross-strait velocity displayed in Fig 6C is easily recognizable in Fig 7B, despite its thickness fluctuations. Periods of enhanced velocity alternate with other periods of reduced or, even, reversed (negative) velocity. The point of interest is the existence of sustained periods of enlarged velocity nearby the interface depth that provides good chances for south-to-north transport. According to Fig 7, they seem to occur preferably in winter (December 2020 to February 2021). Similar velocity and thickness fluctuations are seen in the surface layer, affecting in this case the north-to-south connectivity. Therefore, subinertial variability offers windows of enhanced cross-strait velocity lasting long enough to allow for successful connection between both shores, a result that applies for both directions.

Velocity fluctuations in Fig 7B may have different origins. A correlation study for investigating relationships between the velocity field and external drivers has been done. Absolute velocity values have been used to avoid the unintuitive detail that higher values of negative velocities mean lower intensities and vice-versa. Panels 7A to 7D show the results for along-strait velocity. Since exchanged flows are computed from this component and the response of those flows to external agents is well established [24, 33], these panels are used here as quality tools. They are expected to show confirmation of this response, which in turn will provide support to the conclusions drawn from panels 7E to 7H for the much less studied cross-strait velocity, the one of interest in this work.

#### Meteorologically-induced fluctuations

**Fig 8:**
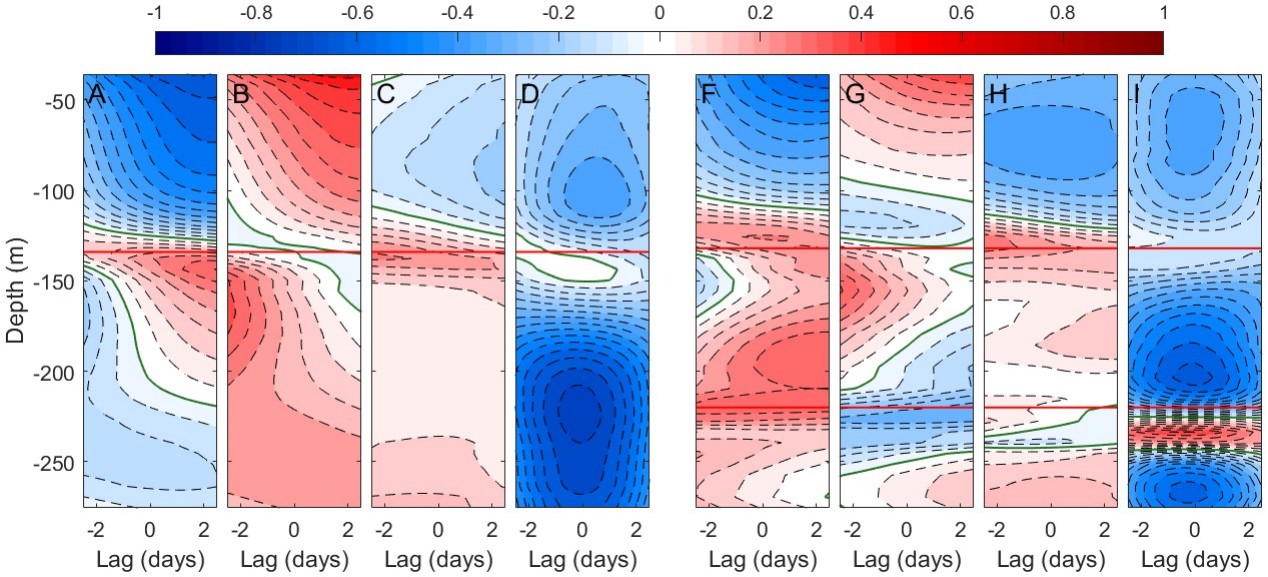
Left panels: contours of lagged correlation as a function of depth between the modulus of along-strait velocity with A) the atmospheric pressure in Palma de Mallorca (see red dot in Fig 1), taken as representative of the Western Mediterranean basin, B) the local zonal component of wind, C) the meridional component of wind and D) the strength of the spring-neap tidal cycle as determined by the envelope displayed in Fig 7A. Contour lines are every 0.05 units of correlation with the zero contour shown in green. Horizontal red line indicates the depth of zero-crossing of the along-strait velocity (see Fig 6C). Right panels: same as left panels but for the cross-strait velocity. Red lines indicate the depths of cross-strait zero velocity. In all cases, positive lags correspond to “forcing” (i.e., atmospheric pressure, wind, strength of the tide) leading the “response” (i.e., the velocity components).

The along-strait velocity is negatively correlated with the atmospheric pressure over the western Mediterranean above the interface and positively below it (Fig 8A). High pressure will reduce the inflow and increase the outflow, giving rise to a barotropic fluctuation of the net flow towards the Atlantic. Westerlies (positive zonal winds) increase the velocity in the upper layer, easterlies (negative zonal winds) diminish it (Fig 8B). Lower layer velocities show positive correlation too, suggesting that wind-stress induces baroclinic fluctuations of the exchange. These results have already been reported [24, 33, 34, 38, 39] and provide indirect support to those inferred from the correlation analysis for cross-strait velocities, which are discussed next.

Focusing on the upper and intermediate layers that extend over the depth range occupied by the interface (Figs 4 and 5), Fig 8F shows negative and positive correlations, respectively, with the modulus of the cross-strait velocity. Therefore, high atmospheric pressure will diminish the southward current in the upper layer and increase the northward one in the intermediate layer. It shares characteristics of its counterpart Fig 8A in the sense that the response is a barotropic fluctuation that shifts the secondary cross-strait circulation towards a situation of decreased surface current. The opposite would happen under low pressures. Zonal winds (Fig 8G) produce positive correlation in the top of the upper layer and in the intermediate layer. Westerlies would increase the velocity in these segments of the water column and enhance the secondary cross-strait circulation. The opposite effect would happen with easterlies. The effect of meridional winds (Fig 8H) recalls the one of atmospheric pressure (Fig 8F). Northward (positive) wind reduces the mean southward cross-strait velocity in the upper layer and increases the northward one in the intermediate layer. The opposite would happen with southward winds. Both results are expectable.

#### The fortnightly tidal cycle

One of the clearer features in Fig 7B is the fortnightly fluctuations of the cross-strait velocity, which reveals their tidal origin. Fig 8I, in fact, shows extended negative correlation with the strength of the tide in the upper and intermediate layers, meaning that the secondary cross-strait circulation is diminished during spring tides and enhanced during neap tides. A similar conclusion is drawn from the negative correlation between the strength of the tide and the modulus of the along-strait velocity in practically the entire water column displayed in Fig 8D. The correlation is particularly high in the lower Mediterranean layer, which implies weaker subinertial, tidal-free currents in spring tide and stronger in neap tide. This somewhat unintuitive result has been discussed in [20, 35], and is ascribed to enhanced mixing during spring tides which diminishes the horizontal density gradient that drives the flow. Fig 8D is encouraging in the sense that depicts a contrasted result that supports the findings for the cross-strait velocity component.

The joint consideration of the outcome of the correlation analysis allows to stress favorable opportunity windows and unfavorable scenarios for cross-strait connections. Regarding meteorological forcing, northward flow within the intermediate layer would increase under high pressure over the western Mediterranean basin and local winds from the southwest. If, moreover, these particular conditions are met during neap tides, the resulting scenario would gather the best conditions for south-to-north connectivity. The opposite would happen under low pressure and local northeasterly in spring tides, which would diminish the chances. As for the upper layer, the southwards flow that favors north-to south connection would increase under low atmospheric pressure and local northwesterly along with neap tides. Chances would decrease with low pressure and southeasterly in spring tides.

## Other requirements for a successful connection

Let us consider one of the shores of the SoG with algae settlements while the other is still free of them. What conditions must be met to achieve a successful colonization of the algae-free shore without human intervention? First of all is the existence of the cross-strait system of flows described in the previous sections. The current to take advantage of depends on which shore holds the initial settlement: should it be the north one, the right current is that at the surface layer, but if the initially colonized is the south shore, the proper current lays in the intermediate layer. In addition to this secondary circulation, the successful colonization requires other set of circumstances that include physiological traits and endurance of algae.

The weak cross-strait flow is able to transport products from a shore to the other only if they are available. For south-to-north transport, for instance, algal fragments must reach the interface, situated 100–150 m below the sea surface in the south, be carried to the north at these depths and, once there, be moved upwards into the illuminated layer to settle and thrive. The journey will take days to weeks and be done in dark conditions. The colonization success will thus depend on whether or not the algae arrive in good conditions for reproduction after that period of weak light or no-light conditions.

### Reaching the right depth for crossing and returning to illuminated layers

Macroalgal strains are attached to rocky illuminated bottom and other type of substrates in the subtidal nearshore zone of the SoG to a maximum observed depth of 40 m [1]. There are also evidences that they are drifting freely well below this depth (Fig 2). They must be torn out from the substrate to provide free spores, propagules or seaweed fragments for transportation. Density of algae and vertical velocity of the flow come into play at this stage. *Rugulopteryx okamurae* is very slightly denser than seawater. Simple experiments performed in calm sea conditions in which thalli were dropped near the surface and allowed to fall freely, yielded sedimentation velocities in the range of 1 to 4 cm·s^-1^, depending on the size and shape of the thalli and on whether they form larger aggregates. Similar experiments carried out under more controlled conditions in the laboratory gave a little broader sedimentation velocity range (from 1 to 6.5 cm·s^-1^) and confirmed its dependence on the shape and size of the specimens. Variations of seawater density within realistic intervals will hardly change that velocity. The small density difference makes the algae be easily re-suspended by wind-induced or current-induced turbulence, carried to the surface and eventually beached on the shores, often in astonishing amounts [1, 5, 15, 40] see Fig 2B.

**Fig 9:**
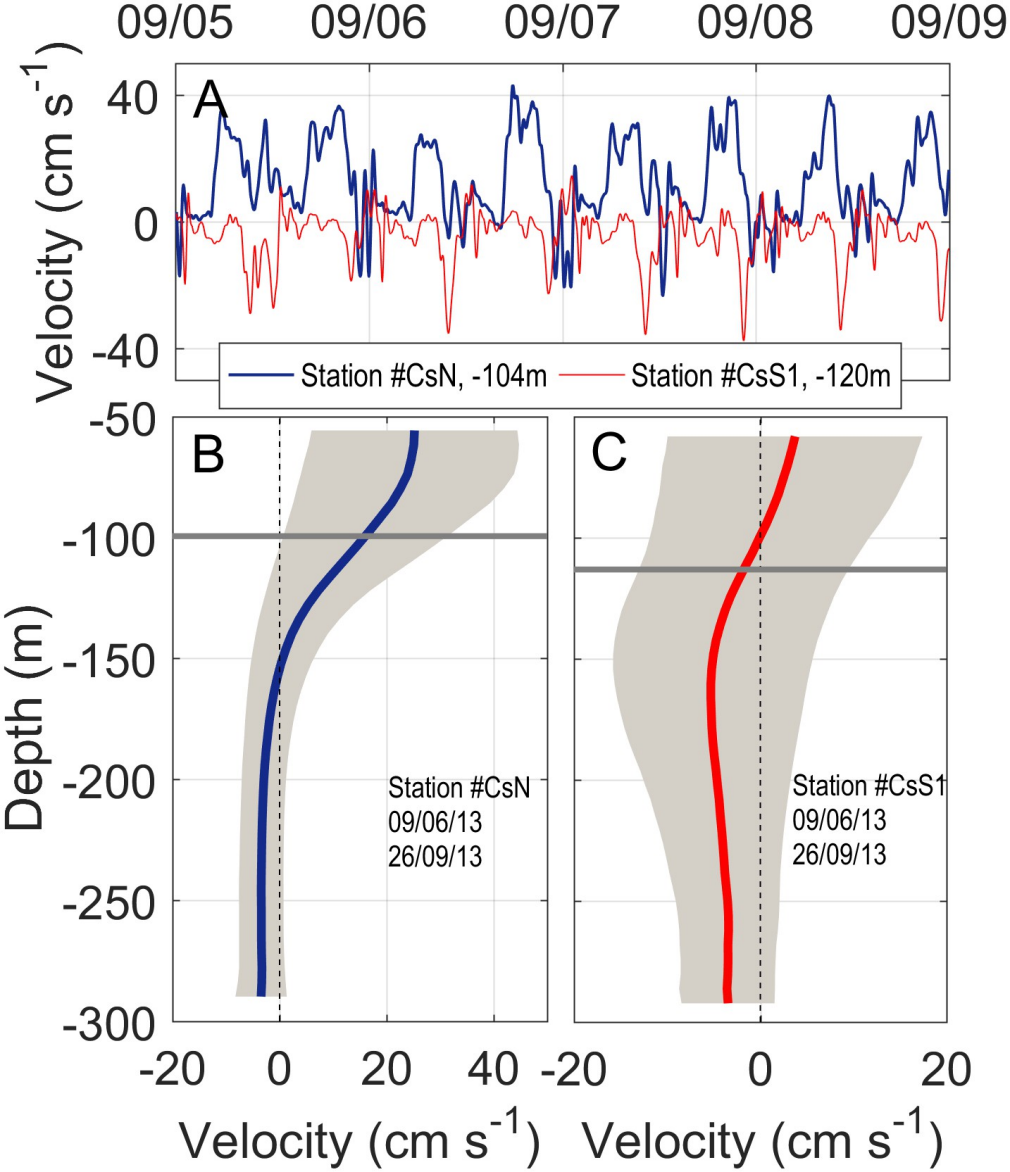
A) Vertical velocity recorded at stations CsN and CsS1 at depths of 104m and 120m, respectively, close to the time-averaged interface depth. Dates on the top axis correspond to September 2013. Velocities were recorded every two minutes and have been smoothed by a 5min cut-off period filter. B) Vertical profile of the time-averaged vertical velocity at station CsN (blue line). Shaded area stretches over ±1std and the horizontal grey line indicates the (mean) interface depth. C) Same as B) but for station CsS1.

Flow-topography interaction in the SoG forms internal hydraulic jumps with high levels of turbulence nearby Camarinal sill [41]. They decay as short-period, large-amplitude internal waves [42] with associated vertical velocities of tens of cm·s^-1^ [43], greater than the measured sedimentation velocity. Fig 9A shows a short time series of vertical velocity near the interface at stations CsN and CsS1 and illustrates the fact. It changes sign and exhibits semidiurnal fluctuations that disclose their tidal origin. Absolute values exceed the sedimentation velocity and can therefore displace algal fragments up or down vertically. Profiles in Fig 8B and 8C suggest that this result applies to the whole water column. However, nearby the interface, downward advection is more likely to occur in the south and upward advection in the north, as it can also be deduced from Fig 9A.

The combination of the very small negative buoyancy of algal fragments and the structure and strength of vertical velocities in the SoG makes it feasible for the algae to reach the suitable depth to be advected across the SoG. They would do it in one direction or the other, depending on the depth range in which they stay (cf., Fig 4). Once on the other side, vertical currents could bring them into the illuminated surface layers and allow them to begin colonizing new environments.

### Keeping the right conditions for spreading

The colonization will depend on the health status the algae arrive with after a relatively long journey made in low light or, even, no light conditions. To gain insight into this issue, *Rugulopteryx okamurae* specimens collected in Tarifa (see Fig 1 for location) were placed in darkness inside a culture chamber of nutrient-rich seawater at 17°C. After 13 days, they showed a considerable health decay, measured as its photosynthetic capacity. However, these specimens were able to recover partially when they were re-exposed to light after the dark period. Even though the initial condition was not fully achieved, they were able to reactivate their metabolism again, thus offering chances for colonizing the new region of arrival. This outcome agrees with other studies. Photosynthetic measurement using *in vivo chlorophyll fluorescence* in algae collected in nets during fish activities between 50-100m depth in the same area gave satisfactory value of electron transport rate as indicator of photosynthetic activity [44]. Also, [18] observed that adult thalli of the species had survival rates between 80–100% after being cultivated in dark conditions for three weeks, depending on the temperature during cultivation, and that these thalli even increased their biomass during this period.

This outcome is not new in the algae realm. Microscopic stages of macroalgae can withstand long periods of darkness and subsequently develop when conditions improve [45–47]. *Aureococcus anaphagefferens* (Pelagophyceae), a heterokont alga responsible for the harmful brown tides, was able to survive for at least 30 days in the dark according to [48]. Otherwise, arctic macroalgae (Laminarians and some Rhodophytes) and different polar phytoplankton can survive during the polar night with up to six-month darkness, when they are unable to photosynthesize. They do it by evolving specific strategies during darkness, like maintaining low metabolic state to decrease the consumption of storage compounds and energy. When light returns after the dark period, they re-initiate the growth [49–51]. The mechanisms involved in survival during darkness are not yet well known for these species, nor for *Rugulopteryx okamurae*. Further studies are required on this. However, the point of interest here is that there are real possibilities of survival during the time interval needed for transportation from one shore to the other.

## Summary and final remarks

Simultaneous appearance of *Rugulopteryx okamurae* in both shores of the SoG few years ago seems highly improbable. A much more likely situation is that the algae settled in one shore, from which it invaded the opposite one. Favorable environmental conditions propitiated its subsequent explosive spreading. Algae capability to colonize any type of hard substrates [15] opens the possibility of accidental crossing attached to ship hulls, fishing nets or other human-mediated mechanisms, but the possibility still remains that it could have managed to cross the SoG taking advantage of hydrodynamic processes without human intervention.

The possibility has been assessed in this study using current meter observations collected in the area, which show layers of no null velocity in the cross-strait direction. Chances for cross-strait transport reduce to the depth range around the interface of null along-strait velocity, located at around 100–150 m depth. Within this range, layers of weak cross-strait currents heading south and north have been identified above and below the interface, respectively. They could be used by nearly-neutral buoyant seaweed fragments for being transported from one shore to the other. Outside of this depth range, possibilities drop null, as particles will be advected beyond the lateral open boundaries of the SoG by the strong inflow and outflow.

The successful connection, however, relies on a chain of circumstantial links. Algae should be ripped out from the seafloor by natural (wind-induced or current-induced turbulence) or human (bottom-trawling nets) agents in the surface illuminated layer at the colonized shore. Their propagules or spores should be displaced offshore in the horizontal and to the depth range of the interface in the vertical. Then they should be transported across the deepest part of the SoG until reaching the opposite continental slope, be raised to the surface and displaced to the shore and, finally, settle and thrive in the new environment. The process will take weeks, much of the time the algae being in extreme low or even no-light conditions. This study argues that each and every of these contingencies, considered one by one, are surmountable. For a single experiment, however, the concatenation of the full sequence of events leading to a successful connection is extremely improbable. Obviously, it is only the massive amount of available specimens for transport that can provide a number of successful connections. Even if this number of connections is reduced, it would be enough to colonize the new ecosystem taking into account the huge adaptability of the algae. The feasibility of this possibility could be tested with the help of very high resolution fully 3D numerical models coupled to high resolution advection schemes. The results of these numerical experiments would either support the possibility of a successful hydrodynamic connection between both shores or, on the contrary, they would provide additional proofs to confirm its high improbability and, therefore, reject it. Such a study is currently in progress.

## Supporting information

S1 File Appendix: Summary of current meter observations. (PDF)

## References

[1] García-Gómez JC, Sempere-Valverde J, González AR, Martínez-Chacón M, Olaya-Ponzone L, Sánchez-Moyano E, et al. From exotic to invasive in record time: The extreme impact of Rugulopteryx okamurae (Dictyotales, Ochrophyta) in the strait of Gibraltar. Science of The Total Environment. 2020 Feb;704:135408. doi: 10.1016/j.scitotenv.2019.135408 31836226

[2] Huang ZG. Marine Species and Their Distributions in China’s Seas. China Ocean Press. Beijing; 1994.

[3] Verlaque M, Steen F, De Clerck O. Rugulopteryx (Dictyotales, Phaeophyceae), a genus recently introduced to the Mediterranean. Phycologia. 2009 Nov;48(6):536–42.

[4] Altamirano-Jeschke M, de la Rosa Álamos J, Martínez Medina FJ. Arribazones de la especie exótica Rugulopteryx okamurae (EY Dawson) I.K. Hwang, W.J. Lee & H.S. Kim (Dictyotales, Ochrophyta) en el Estrecho de Gibraltar: primera cita para el Atlántico y España. 2016. RIUMA. http://hdl.handle.net/10630/12433/

[5] El Aamri F, Idhalla M, Tamsouri MN. Occurrence of the invasive brown seaweed Rugulopteryx okamurae (E.Y.Dawson) I.K.Hwang, W.J.Lee & H.S.Kim (Dictyotales, Phaeophyta) in Morocco (Mediterranean Sea). Mediterranean Fisheries and Aquaculture Research (MedFAR). 2018. 1(2), 92–96.

[6] Navarro-Barranco C, Muñoz-Gómez B., Saiz D, Ros M, Guerra-García JM, Altamirano M, et al. Can invasive habitat-forming species play the same role as native ones? The case of the exotic marine macroalga Rugulopteryx okamurae in the Strait of Gibraltar. Biological Invasions. 2019 Nov 1;21(11):3319–34.

[7] Katsanevakis S. Unpublished Mediterranean records of marine alien and cryptogenic species. BioInvasions Records. 2020;9(2):165–82.

[8] Ashton GV, Zabin CJ, Davidson IC, Ruiz GM. Recreational boats routinely transfer organisms and promote marine bioinvasions. Biological Invasions. 2022 Jan 17;24(4):1083–96.

[9] García-Gómez JC, Florido M, Olaya-Ponzone L, Rey Díaz de Rada J, Donázar-Aramendía I, Chacón M, et al. Monitoring Extreme Impacts of Rugulopteryx okamurae (Dictyotales, Ochrophyta) in El Estrecho Natural Park (Biosphere Reserve). Showing Radical Changes in the Underwater Seascape. Frontiers in Ecology and Evolution. 2021 Apr 15;9.

[10] Ruitton S, Blanfuné A, Boudouresque CF, Guillemain D, Michotey V, Roblet S, et al. Rapid Spread of the Invasive Brown Alga Rugulopteryx okamurae in a National Park in Provence (France, Mediterranean Sea). Water. 2021 Aug 23;13(16):2306.

[11] Faria J, Prestes ACL, Moreu I, Cacabelos E, Martins GM. Dramatic changes in the structure of shallow-water marine benthic communities following the invasion by Rugulopteryx Okamurae (Dictyotales, Ochrophyta) in Azores (Ne Atlantic). Marine Pollution Bulletin. 2022;175:113358. doi: 10.1016/j.marpolbul.2022.113358 35092932

[12] Román Muñoz A, Martín-Taboada M, De la Rosa J, Carmona R, Zanolla M, Altamirano M. La modelación de la distribución de especies como herramienta en la gestión de invasiones biológicas en el medio marino: el caso de Rugulopteryx okamurae (Dictyotaceae, Ochrophyta) en el Mediterráneo. Algas. 2019. 55e: 37–41 (in Spanish).

[13] Mercado JM, Gómez-Jakobsen F, Korbee N, Aviles A, Bonomi-Barufi J, Muñoz M, et al. Analyzing environmental factors that favor the growth of the invasive brown macroalga Rugulopteryx okamurae (Ochrophyta): The probable role of the nutrient excess. Marine Pollution Bulletin. 2022 Jan;174:113315. doi: 10.1016/j.marpolbul.2021.113315 35090297

[14] Hwang IK, Lee WJ, Kim HS, De Clerck O. Taxonomic reappraisal of Dilophus okamurae (Dictyotales, Phaeophyta) from the western Pacific Ocean. Phycologia. 2009 Jan;48(1):1–12.

[15] García-Gómez JC, Sempere-Valverde J, Ostalé-Valriberas E, Martínez M, Olaya-Ponzone L, González AR, et al. Rugulopteryx okamurae (EY Dawson) IK Hwang, WJ Lee and HS Kim (Dictyiotales, Ochrophyta), alga exótica “explosiva” en el estrecho de Gibraltar. Observaciones preliminares de su distribución e impacto. Almoraima. Revista de Estudios Campogibraltareños. 2018. 49, 97–113. ISSN 1133-5319.

[16] Wejnerowski Łukasz, Tümer Orhun Aykut, Aleksandra Pełechata, Rybak M, Dulić T, Jussi Meriluoto, et al. Plankton hitch-hikers on naturalists’ instruments as silent intruders of aquatic ecosystems: current risks and possible prevention. NeoBiota. 2022 May 25;73:193–219.

[17] Hyun B, Shin K, Jang MC, Jang PG, Woo Jung Lee, Park CB, et al. Potential invasions of phytoplankton in ship ballast water at South Korean ports. Marine and Freshwater Research. 2016 Dec 16.

[18] Rosas-Guerrero J, Meco YE, Altamirano M. Could Rugulopteryx okamurae (Dictyotales, Ochrophyta) have been introduced by ballast waters? Algas. 2018. 54, 52.

[19] Bryden HL, Candela J, Kinder TH. Exchange through the Strait of Gibraltar. Progress in Oceanography. 1994 Jan;33(3):201–48.

[20] García-Lafuente J, Vargas JF, Francisco Jorquera Plaza, Sarhan T, Candela J, Burkard Bascheck. Tide at the eastern section of the Strait of Gibraltar. Journal of Geophysical Research. 2000 Jun 15;105(C6):14197–213.

[21] García-Lafuente J, Sanchez-Garrido JC, García A, Hidalgo M, Sammartino S, Laiz R. Biophysical Processes Determining the Connectivity of the Alboran Sea Fish Populations. Springer eBooks. 2021 Jan 1;459–87.

[22] Gill A. Atmosphere-Ocean Dynamics. Academic Press, San Diego, California, USA. 1982. 662 pp.

[23] Garrett C, MacCready P, Rhines PB. Boundary Mixing and Arrested Ekman Layers: Rotating Stratified Flow Near a Sloping Boundary. Annual Review of Fluid Mechanics. 1993 Jan 1;25(1):291–323.

[24] Candela J, Winant CD, Bryden HL. Meteorologically forced subinertial flows through the Strait of Gibraltar. Journal of Geophysical Research. 1989;94(C9):12667.

[25] Garrett C. Frictional processes in straits. Deep-sea Research Part Ii-topical Studies in Oceanography. 2004 Feb 1;51(4–5):393–410.

[26] Pratt LJ, Whitehead JC. Rotating Hydraulics. Atmospheric and oceanographic sciences library. Springer Nature (Netherlands); 2007.

[27] Johnson GC, Ohlsen DJ. Frictionally Modified Rotating Hydraulic Channel Exchange and Ocean Outflows. Journal of Physical Oceanography. 1994 Jan 1.

[28] Baschek B, Send U, Garcia-Lafuente J, Candela J. Transport estimates in the Strait of Gibraltar with a tidal inverse model. Journal of Geophysical Research: Oceans. 2001 Dec 15;106(C12):31033–44.

[29] Sammartino S, Lafuente J, Naranjo C, Garrido S, Sanchez F, Sánchez Román A. Ten years of marine current measurements in E spartel S ill, S trait of G ibraltar. Journal Of Geophysical Research: Oceans. 2015 Sep 1;120(9):6309–28.

[30] García-Lafuente J, Sammartino S, Sánchez Garrido JC, Naranjo C. On the role of the bay of algeciras in the exchange across the strait of Gibraltar. Regional Studies in Marine Science. 2019 May 1.

[31] García-Lafuente J, Bruque Pozas E, Sánchez Garrido JC, Sannino G, Sammartino S. The interface mixing layer and the tidal dynamics at the eastern part of the Strait of Gibraltar. Journal of Marine Systems. 2013 May 1;117–118:31–42.

[32] Nadal I, Sammartino S, Jesús García-Lafuente, Garrido JA, Gil-Herrera J, Hidalgo M, et al. Hydrodynamic connectivity and dispersal patterns of a transboundary species (Pagellus bogaraveo) in the Strait of Gibraltar and adjacent basins. Fisheries Oceanography. 2022 Apr 18.

[33] García Lafuente J. Subinertial variability in the flow through the Strait of Gibraltar. Journal of Geophysical Research. 2002;107(C10).

[34] García Lafuente J, Delgado J, Criado F. Inflow interruption by meteorological forcing in the Strait of Gibraltar. Geophysical Research Letters. 2002 Oct;29(19):20–120–4.

[35] Vargas JM, García-Lafuente J, Candela J, Sánchez AJ. Fortnightly and monthly variability of the exchange through the Strait of Gibraltar. Progress in Oceanography. 2006 Aug;70(2–4):466–85.

[36] García-Lafuente J, Sammartino S, Sánchez-Garrido JC, Naranjo C. Asymmetric baroclinic response to tidal forcing along the main sill of the Strait of Gibraltar inferred from mooring observations. In Velarde, M.G., Tarakanov, R.Y. and Marchenko, V. (editors) The Ocean in motion. Springer Oceanography, Switzerland AG. 2018.

[37] Hersbach H, Bell B, Berrisford P, Biavati G, Horányi A, Muñoz Sabater J, et al. ERA5 hourly data on single levels from 1979 to present (Copernicus Climate Change Service (C3S) Climate Data Store (CDS). 2018.

[38] Peliz A, Teles-Machado A, Marchesiello P, Dubert J, García-Lafuente J. Filament generation off the Strait of Gibraltar in response to Gap winds. Dynamics of Atmospheres and Oceans. 2009 Jan 1;46(1–4):36–45.

[39] Boutov D, Peliz A, Pedro, Soares P, Cardoso RM, Prieto L, et al. Inter-annual variability and long term predictability of exchanges through the Strait of Gibraltar. Global and Planetary Change. 2014 Mar 1;114:23–37.

[40] Altamirano-Jeschke M, de la Rosa J, Gil FJM, Gallego ARM. Prolifera en el Estrecho un alga nunca citada en nuestro litoral de origen asiático, ‘‘Rugulopteryx okamurae” ocupa ya una gran extensión. Quercus. 2017. 374, 32–33.

[41] Wesson JC, Gregg MC. Mixing at Camarinal Sill in the Strait of Gibraltar. Journal of Geophysical Research. 1994;99(C5):9847.

[42] Farmer DM, Armi L, Armi L, Farmer DM. The flow of Atlantic water through the Strait of Gibraltar. Progress in Oceanography. 1988 Jan;21(1):1–103.

[43] Sánchez-Garrido JC, Sannino G, Liberti L, García Lafuente J, Pratt L. Numerical modeling of three-dimensional stratified tidal flow over Camarinal Sill, Strait of Gibraltar. Journal of Geophysical Research. 2011 Dec 17;116(C12).

[44] Figueroa FL, Vega J, Gómez-Valderrama M, Korbee N, Bañares E, Flores-Moya A. Invasión de la especie exótica Rugulopteryx okamurae en Andalucía:I: Estudios preliminares de la actividad fotosintética. Algas. 2020. 56:35–46 (in Spanish).

[45] Leukart P, Klaus Lüning. Minimum spectral light requirements and maximum light levels for long-term germling growth of several red algae from different water depths and a green alga. European Journal of Phycology. 1994 May 1;29(2):103–12.

[46] Santelices B, Aedo D, Hoffmann A. Banks of microscopic forms and survival to darkness of propagules and microscopic stages of macroalgae. Revista Chilena de Historia Natural. 2002 Sep 1;75(3).

[47] Carney LT, Edwards MS. Cryptic Processes in the Sea: A Review of Delayed Development in theMicroscopic Life Stages of Marine Macroalgae. ALGAE. 2006 Jun 30;21(2):161–8.

[48] Popels LC, Hutchins DA. FACTORS AFFECTING DARK SURVIVAL OF THE BROWN TIDE ALGA AUREOCOCCUS ANOPHAGEFFERENS (PELAGOPHYCEAE)1. Journal of Phycology. 2002 Aug;38(4):738–44.

[49] LaCour TG, Morin PI, Sciandra T, Donaher NA, Campbell DA, Ferland J, et al. Decoupling light harvesting, electron transport and carbon fixation during prolonged darkness supports rapid recovery upon re-illumination in the Arctic diatom Chaetoceros neogracilis. Polar Biology. 2019 Oct 1;42(10):1787–99.

[50] Li H, Scheschonk L, Heinrich S, Valentin KU, Harms L, Gernot Glöckner, et al. Transcriptomic Responses to Darkness and the Survival Strategy of the Kelp Saccharina latissima in the Early Polar Night. Frontiers in Marine Science. 2020 Dec 23;7.

[51] Gordillo F, Carmona R, Jiménez C. A Warmer Arctic Compromises Winter Survival of Habitat-Forming Seaweeds. Frontiers in Marine Science. 2022 Jan 13;8.

